# Mini-review: Angiotensin- converting enzyme 1 (ACE1) and the impact for diseases such as Alzheimer’s disease, sarcopenia, cancer, and COVID-19

**DOI:** 10.3389/fragi.2023.1117502

**Published:** 2023-01-23

**Authors:** Valquiria Bueno, Daniela Frasca

**Affiliations:** ^1^ Department of Microbiology Immunology and Parasitology, UNIFESP Federal University of São Paulo, São Paulo, Brazil; ^2^ Department of Immunology, University of Miami, Miami, FL, United States

**Keywords:** ageing, angiotensin-converting enzyme (ACE), Alzheimer’s disease, sarcopenia, cancer, COVID-19

## Abstract

Ageing has been associated with comorbidities, systemic low-grade of inflammation, and immunosenescence. Hypertension is the most common morbidity and anti-hypertensives are used for more than 50%. Angiotensin-converting enzyme 1 inhibitors (ACEi) and angiotensin II receptor blockers (ARB) control blood pressure but also seem to play a role in comorbidities such as Alzheimer’s disease, sarcopenia and cancer. The impact of anti-hypertensives in comorbidities is due to the expression of renin-angiotensin system (RAS) in several tissues and body fluids. Angiotensin-converting enzyme 1 (ACE1) has been linked to oxidative stress, metabolism, and inflammation. The levels and activity of ACE1 are under genetic control and polymorphisms have been correlated with susceptibility to Alzheimer’s disease. In addition, some results found that ACEi and ARB users present delayed cognitive decline and reduced risk of dementia. Regarding to sarcopenia, RAS has been linked to the catabolic and anabolic pathways for muscle mass maintenance. In some studies, older adults using ACEi were highly benefited by exercise training. In cancer, RAS and its products have been shown to play a role since their inhibition in animal models modulates tumor microenvironment and improves the delivery of chemotherapy drugs. Clinically, the incidence of colorectal cancer is reduced in patients using ACEi and ARB. During the pandemic COVID-19 it was found that ACE2 receptor plays a role in the entry of SARS-CoV-2 into the host cell. ACE1 genotypes have been linked to an increased risk for COVID-19 and severe disease. In some studies COVID-19 patients taking ARB or ACEi presented better outcome.

## Introduction

Ageing is a complex process which has been associated with comorbidities, systemic low-grade of inflammation, and changes in the frequency/function of immune cells (immunosenescence) (Bueno et al., 2014; Alves et al., 2018; Alves and Bueno, 2019; Pawelec et al., 2021). A comorbidity with high impact in older individuals is hypertension which can affect 27% of individuals younger than 60 years and 74% of older adults with more than 80 years (Lloyd-Jones et al., 2005). Hypertension in the older adults has been linked to an increased risk of ischemic and hemorrhagic strokes, vascular dementia, Alzheimer’s disease, coronary artery disease, atrial fibrillation, chronic kidney disease and retinal diseases (Perry et al., 2000; Vaccarino et al., 2000; Bulpitt et al., 2003; Rosendorff et al., 2007). Non-pharmacological approaches includes healthy diet, physical activity, non-smoking, avoidance of high intake of alcohol, among others. However, pharmacological interventions can be required, and the benefits on cardiovascular outcomes can be reached by thiazide diuretics, angiotensin-converting enzyme inhibitor (ACEi), angiotensin II receptor blockers (ARB), and calcium channel blocker (CCB). [reviewed in (Oliveros et al., 2020)].

In this mini-review it is not our goal to discuss hypertension and its treatment, since there are excellent articles on the field (Pont and Alhawassi, 2017; Oliveros et al., 2020; Li et al., 2022) and the topic itself would require a new whole article. Instead, our aim is to discuss angiotensin-converting enzyme 1 (ACE1) expression and the possible link with age-related diseases such as Alzheimer’s disease, sarcopenia, cancer, and COVID-19. In addition, we will describe findings on how ACE1 inhibition/blockade can interfere with the outcome of older patients from these conditions. However, it has to be pointed that the benefits observed by the use of anti-hypertensives in some age-related diseases can be linked, at least in part, to the control of high blood pressure.

The angiotensin-converting enzyme 1 (ACE1) is a dipeptidyl carboxylase which can be inhibited leading thus to the reduction of blood pressure *via* the renin-angiotensin system (RAS). In a summary definition, RAS is composed by a vasoconstrictor, pro-inflammatory ACE1/AngII/AT_1_R axis, and a vasodilating anti-inflammatory ACE2/Ang1-7/MasR axis. In addition to the blood pressure control, ACE1 and its peptide substrates affect cardiovascular and renal function, hematopoiesis, reproduction, and the immunity (Bernstein et al., 2012; Bernardi et al., 2016). ACE1 expression has been observed not only in tissues since its soluble form was found in urine, serum, seminal fluid, amniotic fluid, and cerebrospinal fluid (Hooper, 1991). Because of its wide expression in several tissues and body fluids, ACE1 has been associated to some diseases development/progression.

Recently it has been shown, mainly in experimental models, that ACE1 can interfere with several processes in the organism through mechanisms such as oxidative stress, metabolism, and inflammation (de Cavanagh et al., 1999; de Cavanagh et al., 2000; Engeli et al., 2000; de Cavanagh et al., 2001; Godsel et al., 2003; Kuno et al., 2003; Muller et al., 2003). It has also been suggested that ACE1 influences age-related diseases (i.e., Alzheimer’s disease—AD, sarcopenia, cancer) (Hu et al., 1999; Kehoe et al., 1999; Carl-McGrath et al., 2004; Yoshihara et al., 2009; Zhang et al., 2019; MacLachlan et al., 2022). ACE1 levels are under genetic control and many studies have focused on insertions and deletions polymorphisms in intron 16 of the ACE gene as a marker for a functional polymorphism. Many single nucleotide polymorphisms have been detected in the ACE1 gene in recent years and the search for the locations of functional polymorphisms currently represents a topic of extensive investigation. For example, it has been shown that ACE1 polymorphisms are correlated with susceptibility to AD (Hu et al., 1999; Kehoe et al., 1999) but the associated mechanisms are still poorly understood. In addition, it was found recently that in normal ageing, ACE1 expression is increased in brain homogenates and this expression is unchanged in the early stages of AD (MacLachlan et al., 2022) Regarding sarcopenia, Yoshihara et al. (2009) found a weak correlation between ACE1 polymorphism and physical function in aged individuals. In cancer (gastric or colorectal), the same patient presented higher expression of ACE1 in tumor microenvironment than in healthy tissues (Carl-McGrath et al., 2004; Zhang et al., 2019).

In the immune system, our group found that ACE1 (CD143) is expressed not only in all subsets of CD4^+^ and CD8^+^ T cells (naive, central memory, effector memory, and effector memory re-expressing RA), but also in B cells (naive, unswitched memory, switched memory, double negative) and in myeloid cells from PBMC of ageing individuals (Bueno et al., 2022). Within the immune system, ACE1 has been shown to have both beneficial and detrimental effects, due to its role in signalling pathways (Gonzalez-Villalobos et al., 2013). In older adults with significant hypertension it was found that ACE1 inhibitors (ACEi) interfere with cytokines secretion by stimulated T cells in culture (Shasha et al., 1991). In PBMC from controls, it was also observed that ACEi in culture suppress the production of cytokines by monocytes and T cells, confirming the role of ACE in inflammatory signalling pathways (Constantinescu et al., 1998). These findings suggest a role played by ACE1 in the age-related chronic diseases *via* inflammation, but the associated mechanisms are not completely understood yet. It is important to know that the effects of the treatment of individuals with age-associated conditions and/or diseases with ACEi is strictly dose-, host-, and disease-dependent.

**FIGURE 1:**
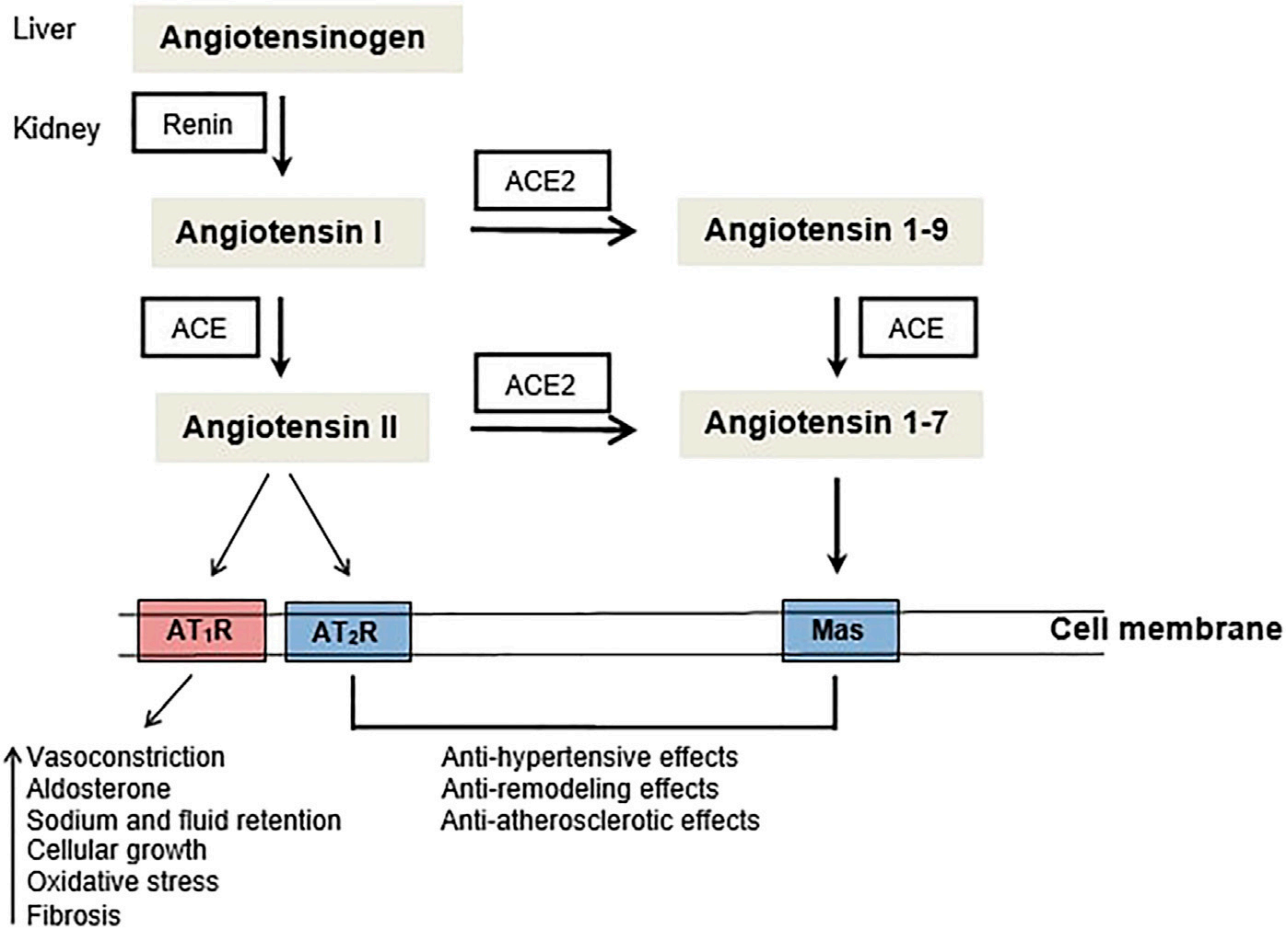
The renin-angiotensin system (RAS): ACE - angiotensin converting enzyme, AT1R - Angiotensin II type 1 receptor, AT2R - Angiotensin II type 2 receptor.

SARS-CoV-2 has been the cause of more than 6 million of deaths worldwide (WHO, 2023) and as the first step for the entry into the host cell the virus binds the human angiotensin-converting enzyme (ACE)2 receptor. Moreover, polymorphisms of ACE1/ACE2 and inhibition/blockade of ACE1 have been associated with patient outcome from COVID-19. Therefore, considering the major impact of infections on aged adults, mainly in those with chronic diseases this mini-review will focus on ACE1 and ACEi and their possible correlation with important age-related conditions such as Alzheimer’s disease, sarcopenia, cancer and SARS-CoV-2 infection. Figure 1.

### ACE1 and cognition/dementia/Alzheimer’s disease

The extension of life expectancy has been related to an increase in cases of dementia and loss of cognitive functions. The most common type of dementia in older adults is AD which has been characterized by reduced synaptic strength, synaptic loss, and neurodegeneration. The hallmark of this disease is the accumulation of senile plaques and neurofibrillary tangles that are respectively associated with changes in the cleavage in amyloid precursor protein (APP)/production of the beta-amyloid (Aβ) and tau protein hyperphosphorylation (Sengoku, 2020). However, it has been proposed that AD clinical symptoms cannot be explained only by the neuropathology of abnormal protein accumulation. As the AD pathogenesis is not completely understood, the current treatments lead to a modest effect in cognition, and therefore it is desirable to develop therapies that can modify the disease development and progress (Cummings et al., 2019). A potential target is the RAS which has been suggested to play a role in AD. ACE inhibitors (ACEi) or blockers of Angiotensin II receptors (ARB) have been shown to delay the cognitive decline and reduce the risk of dementia [reviewed in (Loera-Valencia et al., 2021)].

Yasar et al. (2018) found that cognitively normal older adults (mean age 67.8 years) with elevated ACE1 serum levels at the study entry, presented a worse 1 year evolution in processing speed and work memory suggesting a role played by RAS in neurodegenerative diseases. This hypothesis is reinforced by findings of circulating AngII in regions without blood brain barrier (BBB) and the expression of RAS components in brain. Moreover, ACE1 (acts in the generation of AngII) is expressed in the frontal and temporal lobe and its expression is altered in patients with AD (Arregui et al., 1982; Savaskan et al., 2001; Miners et al., 2008). In opposition, MacLachlan et al. found that in normal ageing, human brain homogenates presented increase of ACE1 and AngII protein levels and decrease of ACE1 activity whereas in early stages of AD it was observed increased ACE1 activity and unchanged ACE1 and AngII protein levels (MacLachlan et al., 2022). In post-mortem brain of older adults (78.5 years) it was found that ACE1 activity correlated inversely with ACE2 activity in AD. Brain homogenates displayed significantly reduced activity of ACE2 which was related to the disease stage. ACE2 activity correlated inversely with total insoluble amyloid β (Aβ) levels, β-secretase activity and protein tau (ptau) load. Authors suggested that the imbalance between classical ACE1 axis and the regulatory ACE2 axis of RAS plays a role in AD pathogenesis (Kehoe et al., 2016). In older adults with memory complaints or AD and no anti-hypertensive treatment, it was found that ACE1 levels and activity in cerebral spinal fluid (CSF) and serum were reduced in patients with AD. Lower ACE1 in CSF correlated with lower CSF Aβ levels, indicating more brain pathology. Reduced ACE1 in CSF was also linked to lower CSF tau and ptau levels (Jochemsen et al., 2014).

Regarding patients using anti-hypertensives, Yasar et al. (2008) evaluated women (70–80 years) for 9 years and observed that the use of ACEi was associated with 59%–85% lower risk of impairment in speed of processing, executive functioning, and verbal memory. In another study, the use of ACEi during 3 years of follow-up showed that anti-hypertensives with BBB crossing status were associated with 65% less decline in instrumental activities of daily living per year compared to other anti-hypertensive drugs. Anti-hypertensives with no BBB crossing status were associated with increased risk of dementia by 20% per year of medicament exposure (Sink et al., 2009). In cognitively normal hypertension-treated older adults (65–84 years and follow-up of 3.5 years) the use of BBB crossing ACEi was associated with a reduced risk of mild cognitive impairment (Solfrizzi et al., 2013). In a meta-analysis (14 cohorts/6 countries) with a total of 12,849 (50–90 years) individuals it was shown that anti-hypertensive BBB crossing drugs were associated with a better memory recall over a follow-up of 3 years (Ho et al., 2021). Fazal et al. (2017) evaluated hypertensive patients using at the time of Alzheimer’s diagnostic centrally acting ACEi (C-ACEi, *n* = 1,207), non-centrally acting ACEi (NC-ACEi, *n* = 143) and 3,910 using neither. Improved cognition was observed in patients using C-ACEi over the first 9 months after diagnosis in comparison with NC-ACEi. Long-term differences were not found in cognition and survival between the groups.

In conclusion, anti-hypertensive medications reduce the risk of cognitive impairment but it remains unclear whether the benefits are reached *via* blood pressure control alone or *via* action on the RAS components. However, Soto et al. (2013) found in patients (mean age 77.8 years) with AD and using ACEi that the slow rate of cognitive decline over 4 years of follow-up was independent of hypertension at baseline or developed subsequently. In addition, human cortical neuron cell line cultured with Losartan displayed decline in the activation of tau kinases, production of p-tau and reactive oxygen species (De Dios et al., 2022) suggesting reduction of neurotoxicity and neuron death which are causes of dementia.

### ACE1 and sarcopenia/exercise

Essential daily activities can be impaired in older adults because of physical limitations. During the aging process muscle mass can be reduced and the remaining muscle may present reduced quality. Sarcomeric proteins, collagen, fibers and myocites relies on the balance between catabolic and anabolic pathways for muscle mass maintenance. In addition, the replacement of muscle fibers by fibroblasts, extracellular matrix proteins, and conective tissue caracterizes the histological quality and quantity of the muscle. The renin-angiotensin system (RAS) modulates all these processes *via* protein turnover, cellular apoptosis, and collagen metabolism (Song et al., 2005; Burks et al., 2011; Cabello-Verrugio et al., 2011; Cabello-Verrugio et al., 2015).

Decreased physical function in the ageing population has been related with disability, loss of independence, higher risk of cardiovascular morbidity and mortality (Newman et al., 2006; Shaw et al., 2006; Studenski et al., 2011). In addition, chronic diseases (i.e., hypertension) could exacerbate the process of sarcopenia in aged individuals (Brown et al., 2000; Nelson et al., 2004). In hypertensive middle-aged patients and also in healthy ageing males it was shown an inverse correlation between blood pressure and ACE1 activity in vastus lateralis muscle suggesting that ACE1 levels in muscle are influenced by hemodynamic factors (Reneland et al., 1999). However, in spite of ACE1 activity identification in vastus lateralis muscle, no correlation was found between these measurements and age (Reneland and Lithell, 1994). The evaluation of ACE1-mRNA transcripts in vastus lateralis muscle by Schaufelberger et al. (1998) found no difference in ACE1-mRNA transcripts between hypertensive patients and control individuals nor differences in ACE1 gene expression was observed due to ACEi treatment. More recently it has been suggested that ACE1 inhibitors (ACEi) could improve physical function not only *via* regulation of blood pressure but also through direct effects on body composition and secreted factors (Carter et al., 2005). In older adults the muscle wasting linked to AngII is due to the decrease of the growth factor 1 (IGF-1) and since the use of ACEi was capable to elevate the levels of IGF-1, it has been suggested a beneficial role played by ACEi (Song et al., 2005; Giovannini et al., 2010). Buford et al. (2012) evaluated older adults (70–89 years old) with mild to moderate functional deficits and found that ACEi users were highly benefited by 12 months exercise training than non-users. In opposition, Sumukadas et al. (2014) observed that in functionally impaired older individuals (*n* = 170, mean age-75 years), ACEi did not enhance the effect of 20 weeks exercise training. In agreement, Kostka et al. (2021) found that even though older patients (79–82 years old) not taking ACEi presented a negative correlation between muscle power/muscle contraction and ACE activity, patients taking ACEi showed no consistent association with handgrip strength, muscle power, muscle contraction velocity, and functional performance. Witham et al. (2014) followed 639 individuals (mean age 65 years) during 4.4 years and observed no difference in grip strength change per year in ACEi users. However, in patients with knee osteoarthritis, a study of 8 years follow-up of 4,295 individuals (mean age 61.2 years) showed that ACEi use (12.8% of participants) was correlated with a reduced risk of frailty (Veronese et al., 2019). Preliminary findings have also indicated beneficial effects of other RAS inhibitors such as ARBs and renin inhibitors, mostly because of their inhibitory effects on local inflammation and oxidative stress.

Sarcopenia diagnostic is complex and should consider not only muscle loss and impaired function, but also how the age-related decline in cognition affects for example grip strength and gait speed. The results from ACEi effects on muscle mass and function are controversial and are probably linked to differences in the enrolled populations, parameters used to assess muscle mass and strength, period of intervention and follow-up, anti-hypertensive used and protocols of physical activity. Further studies are required to support the evidence of ACEi and ARB beneficial effect in preventing sarcopenia.

### ACE1 and cancer

Cancer is highly incident in ageing individuals, and considering that age is a risk factor for hypertension and requires the use of anti-hypertensive drugs, these pharmacological treatments have been studied to determine whether they interfere with cancer development and progression (Ribatti et al., 2007).

Renin-angiotensin system (RAS) and its products have been linked to tumor growth since AT2, the active fraction derived from angiotensin activity is associated to cellular growth, angiogenesis induction, and activation of AT1R receptor. Intracellular signalling pathways of AT1R are linked to fibroblasts growth factor, epidermal growth factor, tumor growth factor (TGF-) beta, platelets-derived growth factor, nitric oxide synthase, protein kinase C, angiopoiethin 2, and metalloprotease [reviewed in (George et al., 2010)]. Experimental studies have linked the RAS to cancer development. In addition, ACEi or ARBs inhibit experimental tumor growth [reviewed in (George et al., 2010)]. Although the antitumoral mechanism associated with ACEi, and ARBs has not been elucidated yet, it has been proposed a role for Ang II-dependent signalling in inhibiting angiogenesis, inflammation, and cellular proliferation (Escobar et al., 2004; Hillers-Ziemer et al., 2022). Inhibition of the RAS pathway, indeed, actively modulates the tumor microenvironment, modifies the tumor stroma, reduces the stiffness of the matrix and ultimately improves the delivery of chemotherapeutic drugs, suggesting that ACEi and ARBs could provide a complementary treatment in cancer patients. A change in the immune milieu, and in the modulation of macrophages, CD8^+^ T cells and T regulatory cells has also been reported (Escobar et al., 2004; Hillers-Ziemer et al., 2022).

In human lung adenocarcinoma, it was observed that genes encoding for ACE and for the angiotensin II receptor were repressed whereas genes encoding for angiotensinogen were overexpressed (Goldstein et al., 2017). In opposition, AGTR1 (encode AT1 angiotensin II receptor) mRNA was increased in estrogen receptor-positive breast cancer and hormone-independent prostate cancer (Rhodes et al., 2009).

Data with aggregated results of randomized controlled trials (RCT) aim to evaluate the possible increased risk of developing cancer in patients using ACEi or ARBs. In five RCT it was observed a discrete increase in the risk of cancer with the use of ARBs. Patients with cancer such as breast, prostate and lung presented a higher occurrence of new lung cancer if receiving ARB (Sipahi et al., 2010). However, in a meta-analysis of 70 RCT (324,168 participants) it was not found any difference in the risk of cancer with ARB or ACEi *versus* placebo (Bangalore et al., 2011). The study of 15 multicenter double-blind RCT with 138,769 participants and a follow-up of 12 months showed no site-specific cancer incidence (lung, breast, prostate) in patients using ARB *versus* controls (Collaboration, 2011). In a systemic review (meta-analysis–12 publications) Datzman et al. (2019) evaluated ARB and carcinogenesis as primary outcome and concluded that there is no correlation between ARB therapy and the increase in the risk of lung cancer. However, a large cohort in Caucasian patients showed an augmented risk of lung cancer (14% after 5 years) associated with ACEi therapy and increase related to years of using the medicaments (Hicks et al., 2018). Studies in patients with advanced non-small cell lung cancer (NSCLC) showed no detrimental effect of RAS-blockers in the patient outcome (Aydiner et al., 2015; Menter et al., 2017).

### ACE1 and colorectal cancer

Colorectal cancer (CRC) is highly incident in older individuals and a significant percentage of patients suffers from cardiovascular diseases. Data from 2005 to 2008 with 12,648 metastatic CRC patients showed that 52% were 65 years old or more and 48.3% were hypertensive. In this study antibiotics and anti-hypertensives were the most used medicaments (61.7% and 49.7% respectively) (Fu et al., 2011). The use of anti-hypertensive drugs (ACEi and ARBs) have been extensively evaluated in CRC because there is more convincing evidence of cancer risk reduction in patients taking these medicaments. A meta-analysis (6 studies, 113,048 patients) performed by Dai et al. (2015) found that the incidence of CRC was significantly reduced in patients using ACEi/ARB than in non-users. Moreover, this study showed that patients using ACEi/ARB presented a better outcome in CRC. In patients with negative CRC colonoscopy, the use of ACEi or ARB (for at least 180 days) was evaluated and correlated with tumor development between 3 and 36 months after the negative diagnosis. In 3 years of follow-up, patients using ACEi/ARB (*n* = 30,856, 61–78 years) showed significantly lower incidence of CRC than non-users (Asgharzadeh et al., 2018). A retrospective study with 13,982 patients (65 years or more) diagnosed with CRC found that the use of ACEi, beta blockers and thiazide diuretics was associated with reduced mortality. It was also found correlation between adherence to therapy (anti-hypertensive) and decreased specific mortality to CRC. The authors suggested that these drugs could be used in addition to the anti-tumor therapy for the treatment of CRC in stages I-III (Cheung et al., 2020; Balkrishnan et al., 2021). Morris et al. (2016) evaluated patients with rectal cancer treated with surgery and adjuvant radiology and found that users of ACEi/ARB presented a significant positive correlation between anti-hypertensive drugs and complete pathological response after therapy. Authors concluded that ACEi/ARB can modulate the tumor response to neoadjuvant therapy em patients with *in situ* rectal cancer in advanced stage. Engineer et al. (2013) found that patients (60–65 years) with stage III CRC, taking ACEi/ARB drugs or beta blockers and treated with chemotherapy or radiotherapy presented decrease in the tumor progression, less hospitalization, and reduced mortality in comparison with non-users. In contrast, Zeman et al. (2022) (*n* = 112, mean 62 years) observed that patients taking ACEi or ARB, and with metastasis in regional lymph node, history of adenocarcinoma, neoadjuvant therapy, and rectal resection presented reduced survival when compared with non-users. The small number of patients and the more severe disease could be the reason for the difference in Zeman’s results.

In summary, the association between the use of anti-hypertensive drugs and the risk and prognosis of cancer remains inconclusive. In colorectal cancer, the benefit of medicaments such as ACEi and ARB are more evident and some authors suggest that these medicaments could be used in association with chemotherapy, radiotherapy, and check-point blockade with the aim to improve the efficacy of the anti-tumor therapy.

### ACE1 and COVID-19

Considering that COVID-19 and other infectious diseases have a major impact in aged adults, mainly in those with chronic diseases (i.e., hypertension and users of ACEi or ARBs) the association between SARS-CoV-2 infection/outcome and renin-angiotensin system (RAS) has been proposed.

On cell surface, SARS-CoV-2 binds the human angiotensin-converting enzyme (ACE)2 receptor and the transmembrane protease (TMPRSS)2 contributes for the fusion between the virus membrane and the cell membrane with subsequent entry of the virus into the host cell. ACE2 and TMPRSS2 are expressed extensively in organs such as lung, heart, kidney, gastrointestinal tract, among others and have been linked to the SARS-CoV-2 widespread infection (Kwenandar et al., 2020; Yuki et al., 2020; Zou et al., 2020; Cao et al., 2021; Hariyanto et al., 2021). Using systematic review with meta-analysis, several studies have shown some level of correlation between polymorphisms of RAS-related genes and increased risk of developing severe COVID-19 (Dieter et al., 2022; Dobrijevic et al., 2022; Gupta et al., 2022; Saengsiwaritt et al., 2022). Yamamoto et al. (2021) in a Medline database search found a higher link between ACE1 DD genotype and severe COVID-19 whereas small studies show that the ACE1 II genotype is a risk factor. Confirming the association between ACE and COVID-19, it was found that patients (*n* = 1,686, mean age 65.6 years) infected with SARS-CoV-2 and taking ARB or ACEi at hospital admission required less use of ventilation and vasopressors compared with non-users. However, this association was observed only for males and authors suggested that sex-based differences in RAS dysregulation may explain these results since males presented higher plasma ACE1 and AngII than females at baseline and early period of admission (Rocheleau et al., 2022). In another study it was evaluated whether the use of ACEi/ARB (outpatient and in-hospital) had any association with COVID-19 mortality. It was found that these medicaments were independently associated with a reduced risk of in-hospital mortality. Moreover, African American patients whose display higher prevalence of ACE D allele and consequent more severe COVID-19, showed a significant reduction in in-hospital mortality (Li et al., 2021).

In summary, there are few results until now, but they suggest that the individual genetic background and the treatment with ACEi or ARB are associated with a better clinical outcome in COVID-19 disease. More studies are needed, but using the findings obtained until now it will be possible to stratify the individual risk for disease severity and maybe to include ACEi or ARB therapy for those patients with higher risk of worst outcome.

### Concluding remarks

ACE1 expression has a wide tissue and body fluids distribution and interferes with several biological processes. During the ageing process, ACE1 expression/activity is changed in several tissues which has been linked to age-related diseases (Alzheimer’s, sarcopenia, cancer). In addition, ACE expression in lymphoid and myeloid cells of older individuals has been recently shown suggesting that ACE can also influence immunity. Old individuals are more likely hypertensive and use angiotensin-converting enzyme inhibitors (ACEi) or angiotensin receptor blockers (ARBs) and these medicaments can somehow interfere with the outcome from age-related diseases. Some results on cognition impairment and dementia have shown a reduced risk on these conditions in individuals using ACEi or ARBs. In contrast, for muscle wasting, muscle power, sarcopenia and frailty there are contradictory results regarding the benefit of using ACEi or ARBs. In carcinogenesis, the dual colorectal cancer CRC - ACEi or ARBs has been widely studied since the benefits of these drugs for the patient outcome are reported in several clinical trials. In SARS-CoV2 infection the virus entry into the host cells relies on ACE2 expression and some preliminary results show the better outcome of patients taking ACEi or ARB. The precise mechanisms that link the use ACEi or ARBs and age-related diseases are unknown yet but it is possible to stratify each patient risk for disease development/progression and in addition to evaluate ACE polymorphism. Data from each patient could be used to decide whether or not to include ACEi or ARB to other therapies (precision medicine).
